# An unusual cause of fatal pulmonary embolism: A case report

**DOI:** 10.1016/j.rmcr.2025.102303

**Published:** 2025-10-17

**Authors:** Nino Cmor, Eva Dora, Martina Zitko, Tomaz Kocjan, Mitja Lainscak

**Affiliations:** a Division of Cardiology, General Hospital Murska Sobota, Murska Sobota, Slovenia; b Department of Vascular Diseases, University Medical Centre Ljubljana, Ljubljana, Slovenia; c Department of Endocrinology, Diabetes and Metabolic Diseases, University Medical Centre Ljubljana, Ljubljana, Slovenia; d Faculty of Medicine, University of Ljubljana, Ljubljana, Slovenia

**Keywords:** Case report, Adrenal tumour, Inferior vena cava thrombosis, Pulmonary embolism, Echocardiography, Pulmonary adenocarcinoma

## Abstract

Adrenal tumours invading the inferior vena cava (IVC) are rare but can cause life-threatening complications such as pulmonary embolism. Early recognition, rapid diagnosis, and prompt imaging—including point-of-care ultrasonography (POCUS), echocardiography, and advanced modalities like CT and PET-CT—are essential for optimal management. We present a patient with pulmonary adenocarcinoma and secondary adrenal tumour that extended into IVC and caused fatal tumour thrombus pulmonary embolism.

## 1 Introduction

Adrenal tumours extending into inferior vena cava (IVC) present considerable clinical challenges due to their rarity and potential for severe complications [[1], [2], [3], [4]]. This rare condition involves tumour thrombus infiltration from the adrenal gland into the IVC, sometimes reaching cardiac chambers, and can lead to pulmonary embolism (PE) [5]. Symptoms like dyspnoea and chest pain prompt for diagnosis, with emergency room imaging (e.g. point of care ultrasonography - POCUS, echocardiography) having a central role in visualizing thrombus, its extension and potential cardiac involvement [[6], [7], [8], [9]]. We describe a case of a secondary adrenal tumour extending into IVC diagnosed by echocardiography.

## 2 Case report

A 45-year-old man, without relevant medical history, presented to the emergency department with pain under the right rib cage. The pain had persisted for three weeks and worsened with movement, leading him to initially suspect a muscle strain. Three days prior, he noticed swelling in both legs and began experiencing dyspnoea during exercise. He also reported an unintentional weight loss of 8 kg over the last three months.

Clinical examination revealed tenderness on palpation under the right rib cage and bilateral leg oedema. A 12-lead ECG showed no abnormalities, and the chest X-ray was within normal limits. Laboratory findings were significant for elevated levels of alkaline phosphatase (ALP = 18.82 μkat/L; reference value 0,67-2,15 μkat/L) and gamma-glutamyl transferase (GGT = 5.54 μkat/L; reference value < 0,92 μkat/L), with normal bilirubin levels and hypoalbuminemia (albumin = 25 g/L).

**Fig. 1:**
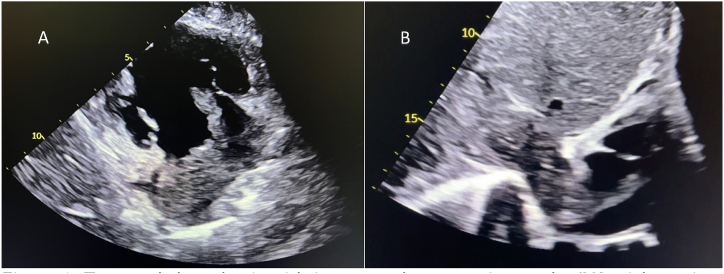
Tumoural thrombosis with intravascular extension to the IVC, right atrium and ventricle on bedside echocardiography. A – parasternal long axis/right ventricular inflow view B- subxiphoid view.

**Fig. 2:**
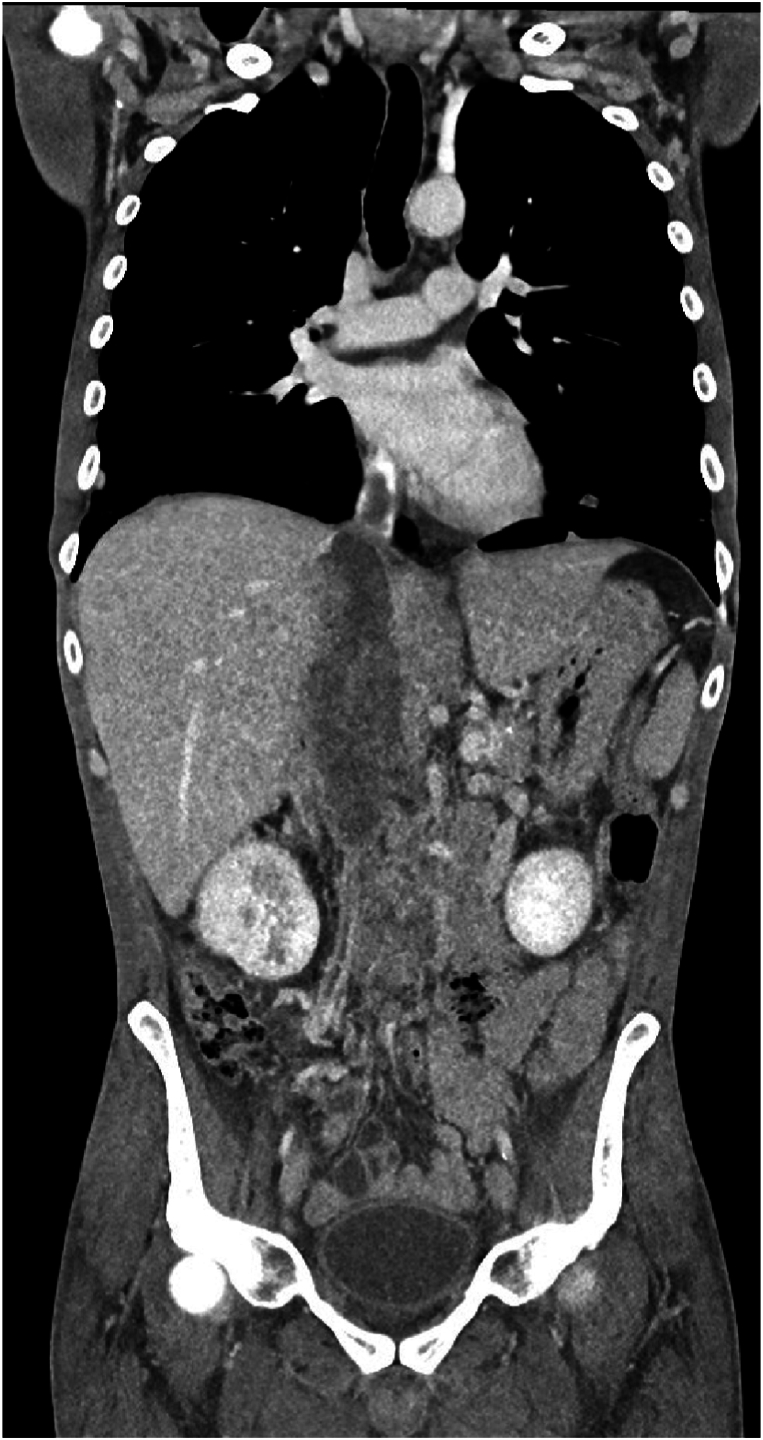
A contrast-enhanced computed tomography scan of the abdomen and thorax: A large tumorous formation of the right adrenal gland, with thrombus extending into the IVC and right atrium.

POCUS showed a right atrial mass with extension into the right ventricle and an obliterated IVC (Fig. 1A–B/VIDEO 1). A contrast-enhanced computed tomography (CT) scan of the abdomen and thorax revealed a 107 × 84 mm large tumorous formation of the right adrenal gland, presumably an adrenocortical carcinoma, with thrombus extending into the IVC and further into both common iliac veins, the right renal vein, the middle hepatic vein, and into the right atrium. Additionally, a lesion was found in the upper right pulmonary lobe, with enlarged right hilar lymph nodes (Fig. 2). CT angiography of the pulmonary arteries revealed a segmental PE.

The following are the Supplementary data related to this article:

Supplementary data related to this article can be found online at https://doi.org/10.1016/j.rmcr.2025.102303.

Multimedia Component 1 Multimedia Component 1

Subcutaneous nadroparin was initiated, and the patient was transferred to a tertiary care hospital for diagnostic assessment. Expanded hormonal testing to exclude a functional adrenal mass (1 mg dexamethasone suppression test, aldosterone, plasma renin activity, 17-OH progesterone, dehydroepiandrosterone sulphate, androstenedione, estradiol, plasma metanephrines and normetanephrines) was normal.

**Fig. 3:**
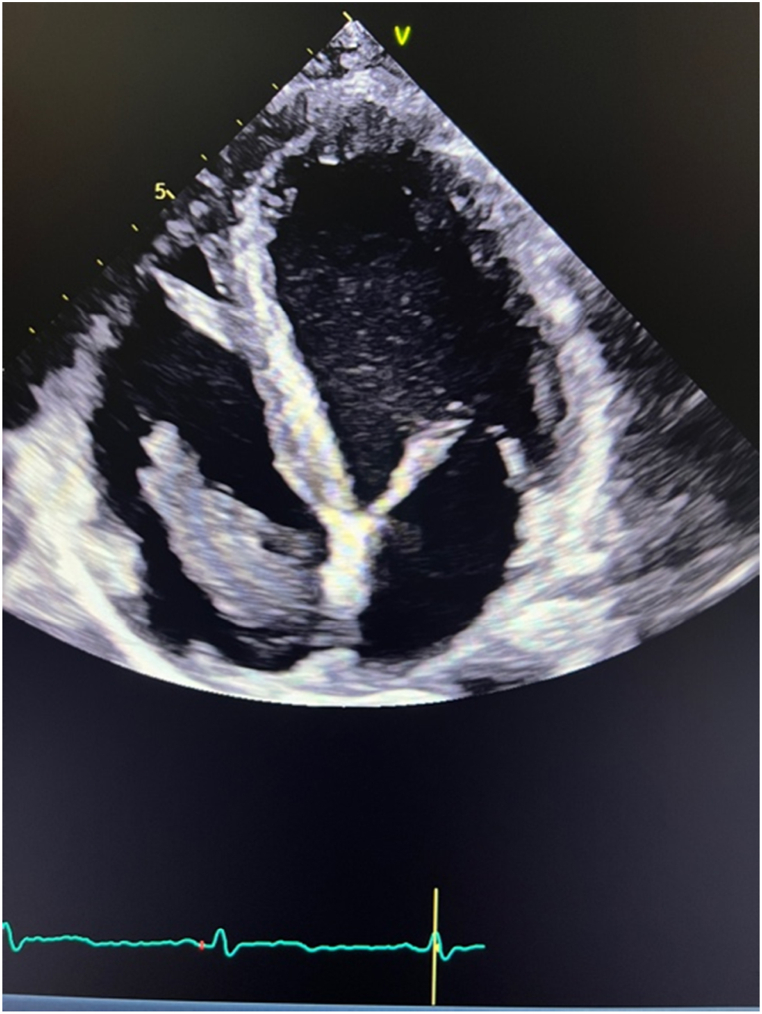
Tumoral thrombosis in right atrium and ventricle with a normal left and right ventricular function on echocardiography.

A comprehensive echocardiography showed a hypoechogenic mass extending from the IVC into the right atrium, prolapsing into the right ventricle, without tricuspid valve dysfunction (Fig. 3/VIDEO 2–4). After excluding a pheochromocytoma, a biopsy of the tumour was performed. Histopathology report diagnosed a poorly differentiated pulmonary adenocarcinoma.

**Fig. 4:**
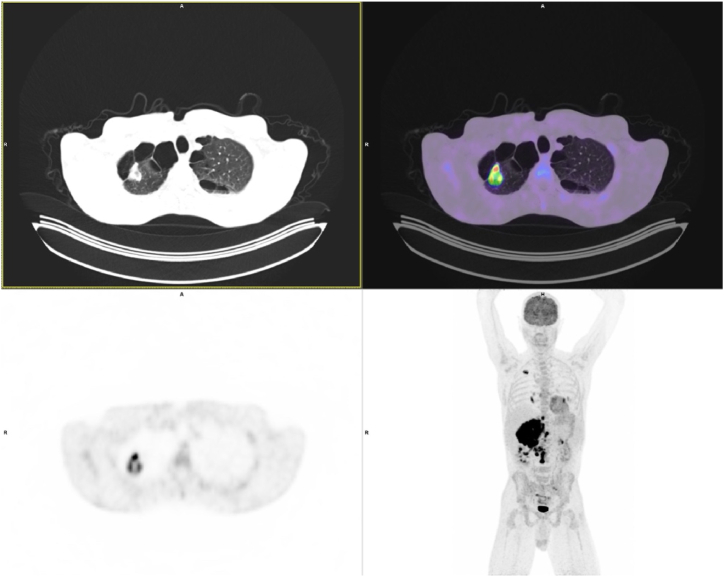
Whole-body 18F-fluorodeoxyglucose positron emission tomography-computed tomography: a hypermetabolic tumour in the right upper lung lobe, with signs of metastatic spread to ipsilateral hilar lymph nodes, ipsilateral and contralateral mediastinal lymph nodes, abdominal lymph nodes, and the right adrenal gland with intravascular extension into the IVC.

The following are the Supplementary data related to this article:

A whole-body 18F-fluorodeoxyglucose positron emission tomography-computed tomography (PET/CT) scan revealed a hypermetabolic tumour in the right upper lung lobe, with signs of metastatic spread to ipsilateral hilar lymph nodes, ipsilateral and contralateral mediastinal lymph nodes, abdominal lymph nodes, and the right adrenal gland with intravascular extension into the IVC (Fig. 4).

Based on comprehensive diagnostic workup, the patient was diagnosed with pulmonary adenocarcinoma with metastatic spread to the right adrenal gland and intravascular extension to the IVC and right atrium. A multidisciplinary team recommended immunotherapy for the primary tumour and palliative radiotherapy of secondary tumour. Due to advanced tumour stage surgical treatment was not seen as a primary option. Patient was discharged after 44 days on subcutaneous nadroparin and analgesics with an outpatient management plan.

Pembrolizumab was initiated and patient had regular radiotherapy of secondary tumour, as planned. During follow-up, liver enzymes normalized, and he was switched to oral anticoagulation with edoxaban. A CT scan six months later demonstrated a reduction of both primary and secondary tumour, along with reduction of tumour thrombus in the IVC while right atrial thrombus was larger. No PE was seen and he was switched back to subcutaneous nadroparin. During regular follow up visit 8 months post discharge patient felt better, had no specific complaints and he gained weight; nadroparin was discontinued because of new-onset thrombocytopenia.

**Fig. 5:**
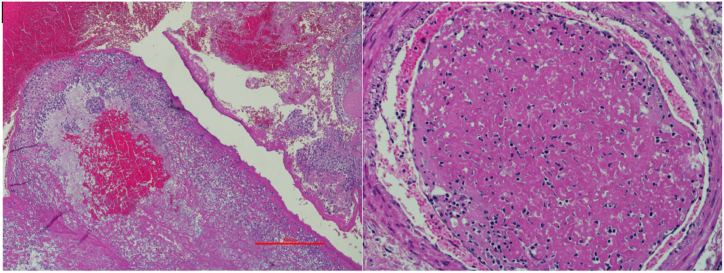
The histologic specimen of the patient's massive pulmonary embolism, which was histologically characterised as tumour thrombi.

Two days after last follow-up visit, he collapsed at home; his partner started resuscitation and called the emergency team. On arrival, he was in asystolic cardiac arrest; despite prolonged and complete resuscitation the patient died. Post-mortem examination confirmed the primary and secondary tumour, and massive PE in proximal left and right pulmonary artery. Emboli were histologically characterised as tumour thrombi (Fig. 5).

## 3 Discussion

Primary and secondary adrenal tumours extending into IVC present significant diagnostic and therapeutic challenges due to their rarity and potential for severe complications [[1], [2], [3], [4]]. These tumours encompass various neoplasms, including adrenocortical carcinomas (ACCs), pheochromocytomas, and metastatic tumours [1]. According to Chesson et al., from 1952 to 2000, there were 105 reported cases of histologically confirmed adrenal tumours involving the IVC, consisting of 78 ACCs, 16 pheochromocytomas, 3 neuroblastomas, and others [2]. Secondary tumours involving the IVC are extremely rare and to the best of authors knowledge, this is the first report of pulmonary adenocarcinoma with secondary adrenal gland involvement that extended into the IVC and right heart.

Patients often present with symptoms consistent with inferior vena cava syndrome, such as abdominal pain, weight loss, dyspnoea, chest pain, and leg swelling [[1], [2], [3], [4]]. In our case, the patient presented with significant leg swelling and dyspnoea, raising suspicion of heart failure. POCUS was performed in the emergency room and was the key for further evaluation [9]. The findings confirmed the thrombus in the IVC, right atrium and a PE. Major left and right ventricular dysfunction were excluded. Nadroparin was initiated as it was not possible to differentiate the thrombus aetiology only by emergency imaging.

The management of adrenal gland tumours with IVC extension requires a multidisciplinary approach [1]. Anticoagulation therapy is a routine early in the patient management due to increased risk of venous thromboembolism [1,5], and should be maintained once the final diagnosis is made. Our patient had a secondary adrenal gland tumour, which according to the guidelines is a contraindication for surgery [1]. Therefore, radiotherapy and immunotherapy in an outpatient setting, along with a close follow-up, pain management and palliative service was organized.

During follow-up, the patient's performance improved, and the malignant disease was in regression. Due to new-onset thrombocytopenia, anticoagulation therapy was discontinued as per guidelines [10]. Two days later, he experienced fatal PE. In the context of this patient, the key question was the embolism origin, with two main potential sources: tumour thrombus or venous thrombus. Post-mortem resolved this dilemma and confirmed massive pulmonary embolism due to tumour thrombi. The cause of death in this young male patient with advanced malignant disease may have been preventable but specific treatment, surgical removal of secondary tumour, is not in line with the current guidelines [1]. Because the guidelines provide general advice that must be tailored to each patient, one could argue that repeated multidisciplinary consultations may be necessary to reassess the patient's condition and weigh the benefits and risks of specific treatment procedures. It is also possible that the oncologic therapy itself mobilized the tumor thrombus, contributing to the development of PE. Therefore, regular, individualized follow-up assessments and reevaluation of the management plan should be implemented for patients with a young biological age and good overall performance status to prevent premature and potentially fatal complications, as illustrated in this case.

## 4 Conclusion

This case promotes crucial role of multimodal imaging and multidisciplinary teamwork to optimize management of adrenal tumours with IVC extension. Additionally, we highlight patient-oriented evaluation of potentially preventable premature death due to tumour pulmonary embolism. Although surgical removal of metastatic adrenal tumours is not guideline-recommended, this could be challenged in patients who respond well to specific therapy, have good performance status and longer than average expected survival. Authors are proponents of regular multidisciplinary consultations to reassess treatment options which may prevent fatal intravascular complications.

## CRediT authorship contribution statement

**Nino Cmor:** Writing – review & editing, Writing – original draft, Conceptualization. **Eva Dora:** Writing – review & editing. **Martina Zitko:** Writing – review & editing. **Tomaz Kocjan:** Writing – review & editing. **Mitja Lainscak:** Writing – review & editing, Conceptualization.

## Declaration of competing interest

The authors declare that they have no known competing financial interests or personal relationships that could have appeared to influence the work reported in this paper.
